# Explicit and Implicit Responses of Seeing Own vs. Others’ Emotions: An Electromyographic Study on the Neurophysiological and Cognitive Basis of the Self-Mirroring Technique

**DOI:** 10.3389/fpsyg.2020.00433

**Published:** 2020-03-31

**Authors:** Alessandra Vergallito, Giulia Mattavelli, Emanuele Lo Gerfo, Stefano Anzani, Viola Rovagnati, Maurizio Speciale, Piergiuseppe Vinai, Paolo Vinai, Luisa Vinai, Leonor J. Romero Lauro

**Affiliations:** ^1^Dipartimento di Psicologia, Università degli Studi di Milano-Bicocca, Milan, Italy; ^2^NETS, Scuola Universitaria Superiore IUSS, Pavia, Italy; ^3^Clinical Psychology Service of Mediterranean Institute for Transplantation and Advanced Specialized Therapies (IRCSS IsMeTT), Palermo, Italy; ^4^Department of Neuroscience, Imaging and Clinical Sciences, “G. d’Annunzio” University of Chieti–Pescara, Chieti, Italy; ^5^“GNOSIS” Research and Psychotherapy Group, Mondovì, Italy; ^6^Studi Cognitivi, Cognitive Psychotherapy School and Research Center, Milan, Italy; ^7^Psicologia Scientifica – Centro di Ricerca e Promozione Sociale, Milan, Italy; ^8^Pôle de Psychiatrie et Psychothérapie du Centre Hospitalier du Valais Romand, Monthey, Switzerland

**Keywords:** facial mimicry, Self-Mirroring Technique, facial expression, EMG, emotion recognition

## Abstract

Facial mimicry is described by embodied cognition theories as a human mirror system-based neural mechanism underpinning emotion recognition. This could play a critical role in the Self-Mirroring Technique (SMT), a method used in psychotherapy to foster patients’ emotion recognition by showing them a video of their own face recorded during an emotionally salient moment. However, dissociation in facial mimicry during the perception of own and others’ emotions has not been investigated so far. In the present study, we measured electromyographic (EMG) activity from three facial muscles, namely, the zygomaticus major (ZM), the corrugator supercilii (CS), and the levator labii superioris (LLS) while participants were presented with video clips depicting their own face or other unknown faces expressing anger, happiness, sadness, disgust, fear, or a neutral emotion. The results showed that processing self vs. other expressions differently modulated emotion perception at the explicit and implicit muscular levels. Participants were significantly less accurate in recognizing their own vs. others’ neutral expressions and rated fearful, disgusted, and neutral expressions as more arousing in the self condition than in the other condition. Even facial EMG evidenced different activations for self vs. other facial expressions. Increased activation of the ZM muscle was found in the self condition compared to the other condition for anger and disgust. Activation of the CS muscle was lower for self than for others’ expressions during processing a happy, sad, fearful, or neutral emotion. Finally, the LLS muscle showed increased activation in the self condition compared to the other condition for sad and fearful expressions but increased activation in the other condition compared to the self condition for happy and neutral expressions. Taken together, our complex pattern of results suggests a dissociation at both the explicit and implicit levels in emotional processing of self vs. other emotions that, in the light of the Emotion in Context view, suggests that STM effectiveness is primarily due to a contextual–interpretative process that occurs *before* that facial mimicry takes place.

## Introduction

Difficulty in accessing and recognizing emotions is a primary problem in many psychiatric and psychological diseases (Keltner and Kring, 1998; Tse and Bond, 2004), such as major depression (see Leppänen, 2006; Bourke et al., 2010 for a review), anxiety (Zeitlin and McNally, 1993; Silvia et al., 2006; Demenescu et al., 2010; Karukivi et al., 2010), and eating (Schmidt et al., 1993; Corcos et al., 2000; Speranza et al., 2007; Harrison et al., 2010) and personality (Domes et al., 2009; Loas, 2012; Loas et al., 2012) disorders. Indeed, alexithymia, which is a clinical condition characterized by difficulty in identifying and describing emotions, is present in over 50% of patients seeking psychological help (Sifneos, 1973; Kojima, 2012). This condition not only contributes to the emergence of symptoms (Güleç et al., 2013) but also influences the psychotherapeutic process (Ogrodniczuk et al., 2004; Leweke et al., 2009) and patients’ compliance to treatments (Speranza et al., 2011).

Therefore, improving patients’ ability to recognize and elaborate on their own emotions is a central goal of psychotherapy, regardless of the specific orientation. In the Cognitive Behavioral Therapy (CBT), for instance, achieving emotional awareness is a primary and fundamental step, since the core of the therapeutic process relies on instructing patients to monitor their feelings and thoughts in different situations, unveiling the connection between thoughts, emotions, and actions. Therefore, patients with poor introspective and self-reflective abilities might encounter great difficulty in the proper detection, description, and naming of their emotional experience. Trying to provide a solution for this issue, Vinai et al. (2015) created a video-based methodology called the Self-Mirroring Technique (SMT). SMT has been developed in the clinical setting and can be used as a coadjutant in different psychotherapeutic approaches. It is based on the audio-visual recording of the therapeutic session, with the aim of showing to the patients the emotions conveyed by their facial expressions. The procedure consists of asking patients to recall an emotionally significant event in their lives while their face is video recorded. Immediately after recall, the psychotherapist shows the patients their own video on the screen and again videos their face. The clinician then shows the patients the effects of seeing their own emotions (for more details on the clinical protocol, see Vinai et al., 2015). The observation of and listening to the video recordings are useful for patients to observe their own thoughts and emotions from an external position and has the effect of increasing their ability to recognize own emotions (Vinai et al., 2015), thus enhancing metacognition (Lorenzini et al., 2006).

Previous studies have reported the effectiveness of SMT in the psychotherapeutic setting (Vinai et al., 2016; Frau et al., 2018). However, the cognitive and neurophysiological mechanisms underlying this success are not completely clear. On the one hand, authors have suggested that observing a video depicting their own emotional expressions may help patients to recognize their own emotions by employing the innate system, which is typically used to understand others’ emotions (Frau et al., 2018), thus improving the patients’ poor ability in the self-reflective and introspective functions. Another possible—and not mutually exclusive—hypothesis suggests that giving patients the opportunity to view their face while listening to their words might allow them to add new—supplementary—information to their previous knowledge, thus facilitating the transition from an unidentified emotion to emotional awareness. Moreover, viewing themselves while feeling an emotion might elicit another emotion, which could be helpful not only for understanding their own emotional state but also for accepting or managing it. For instance, the observation of their own face expressing sadness might induce self-compassion (e.g., Petrocchi et al., 2017).

The STM foundation can be explored more thoroughly by referring to the phenomenon of *facial* (or emotional) *mimicry*, which has been defined as “*the imitation of emotional (facial) expressions of another person*” (Hess and Fischer, 2013; Hess and Fischer, 2017). In the literature, facial mimicry has been investigated by presenting participants with emotional stimuli and recording the activity of specific facial muscles, typically through electromyography (EMG; e.g., Dimberg, 1982; Larsen et al., 2003), and less frequently using the Facial Action Coding System^1^ (Ekman and Friesen, 1978; Murata et al., 2016).

In EMG studies, participants are typically presented with static pictures (e.g., Dimberg and Thunberg, 1998; Scarpazza et al., 2018) and more rarely with dynamic stimuli (Sato et al., 2008; Rymarczyk et al., 2011) and face-to-face interactions (Künecke et al., 2017). Crucially, most studies focused on two emotions, namely, anger and happiness, and rarely included other discrete emotions (see Hess and Fischer, 2013, for a review). The most robust pattern emerging across the studies is the double dissociation between *corrugator supercilii* (CS) and *zygomaticus major* (ZM) muscle activity, which is dependent on the expression valence. Indeed, the presentation of angry—negative valence—faces increased activity in the CS, namely, the muscle used to approximate the eyebrows when frowning, while happy—positive valence—expressions induced higher EMG activity in the ZM, which is the muscle used when smiling, combined with decreased activity in the CS (Dimberg and Thunberg, 1998; De Wied et al., 2006). Other emotions have been less systematically investigated, and weaker link between emotions and muscular activation have been established (see Hess and Fischer, 2013). For example, increased activity in the CS has also been linked to sadness (e.g., Weyers et al., 2009—neutrally primed group; Hess and Blairy, 2001) and fear (e.g., Van Der Schalk et al., 2011), while these patterns did not emerge in other studies (e.g., Lundqvist, 1995; Oberman et al., 2009). Increased activity in the levator labii superioris (LLS) has sometimes been reported in response to a disgust expression (Lundqvist and Dimberg, 1995), but not in a consistent way (Hess and Blairy, 2001).

Noteworthily, over the years, different proposals have addressed the interpretation of facial mimicry, generally focusing on different aspects of the phenomenon and—respective to our aim of deepening STM functioning—leading to different predictions.

According to the *embodied hypothesis*, viewing an emotional expression triggers activity in the same brain regions and peripheral efferent involved in the execution of similar expressions, thus eliciting—through a feedback process—the corresponding emotional state in the mimicker, a process known as *sensorimotor simulation* (see for a recent review Wood et al., 2016). Sensorimotor simulation can lead to a motor output—though overt mimicry is not a necessary component (Goldman and Sripada, 2005)—thus facilitating emotion recognition (Stel and Van Knippenberg, 2008; Neal and Chartrand, 2011; but see Hess and Fischer, 2017 for a critical review) and understanding (Niedenthal, 2007; Oberman and Ramachandran, 2007; Bastiaansen et al., 2009; Niedenthal et al., 2010; Gallese and Sinigaglia, 2011).

This interpretation is in line with the classical view, named by Hess and Fischer (2013) the *Matched Motor Hypothesis*. According to this view, facial mimicry is an automatic motor response and is independent of the intentions of both the expresser and the observer (Chartrand and Bargh, 1999; Preston and De Waal, 2002; for a review, see Hess and Fischer, 2013).

However, previous evidence suggested that mimicry can be influenced by different contextual cues, such as the type of emotion and the expresser’s and observer’s identity, relationship, and emotional state. These points are considered by an alternative account of facial mimicry known as the *Emotion Mimicry in Context view* (Hess and Fischer, 2013, 2017). Its authors suggested that facial expressions are *intrinsically meaningful*; indeed, they convey information about the feelings, thoughts, and intentions of others. The authors suggested that facial mimicry cannot be based merely on a perception-behavior link; rather, it requires the interpretation of the intention of a specific emotional stimulus in each context.

Following the evidence described so far, we created an experimental setting to investigate the cognitive and neurophysiological mechanisms underlying STM functioning by exploring how healthy participants process others’ and their own emotional facial expressions both at an implicit and explicit level. To do so, we created a two-step study. In the preliminary experiment, we presented film excerpts (Schaefer et al., 2010) to induce specific discrete emotions in participants. Participants were video recorded during film viewing in order to create ecologic and dynamic facial videos, which were then validated by a group of 15 judges and were used as stimuli in the “others” condition of the main experiment. Following the same procedure, in the main experiment, we first recorded participants’ faces while they watched the same movie excerpts, thus creating the dynamic stimuli belonging to the “self” condition, and then measured EMG facial activity during the observation of video depicting their own vs. others’ facial expressions. Emotion recognition accuracy, valence, and arousal ratings were collected during the experiment as explicit measures.

Following the Motor Match Hypothesis prediction, we hypothesize that the EMG activity elicited by the different emotions will be congruent with the muscles involved in expressing the same emotion, confirming the specificity of sensorimotor simulation. Indeed, according to the theory, facial mimicry is an automatic match motor response that is not influenced by contextual or interpretative information. Within this framework, the beneficial effect of STM in the therapeutic process might be primarily caused by a more primitive and implicit sensorimotor/embodied re-experience of the seen expressions, which promotes the transition to a more mature and explicit self-reflective and interpretative ability, leading to the possibility of becoming aware of own emotions.

Conversely, finding a dissociation between the emotion seen and the corresponding EMG activity and/or the emotion expressed by own vs. others’ facial expressions would support the Emotion Mimicry in Context view, thus corroborating the idea that a contextual–interpretative process occurs *before* facial mimicry takes place (Hess and Fischer, 2014) and is a prerequisite for STM effectiveness. In this case, the clinical efficacy of SMT could be primarily due to the supplementary information provided by observing their own emotions and hearing their own words, which helps patients to integrate their emotional experience at a richer and multisensory level.

Of course, the two mechanisms should be considered as not mutually exclusive and can coexist in the same patients during specific events, emotions, or moments of their lives.

## Materials and Methods

### Preliminary Experiment: Stimuli Preparation and Validation

#### Phase 1: Stimuli Preparation

In the first phase, we recorded the faces of 15 volunteers while they were viewing 12 emotion-eliciting film excepts. The aim of this phase was twofold. First, we wanted to validate the efficacy of the selected movie clips in eliciting specific emotions. Second, we needed to create stimuli of dynamic and ecological facial expressions for the main experiment.

##### Participants

Fifteen volunteers (six males, M age = 23.1, range 20–30 years old) took part in the experiment in exchange for course credits. Participants were Caucasian, without beard or mustache, with normal or contact lens-corrected eyesight. Participants were naïve to the purpose of the study.

The entire study was approved by the local ethical committee, and participants were treated in accordance with the Declaration of Helsinki. All participants provided written informed consent to be recorded during the experiment and granted their authorization for the use of recordings for scientific purposes.

##### Procedure and analysis

Participants took part in the experiment individually. They sat in an artificially illuminated room at 60 cm from a 15.7” laptop monitor on which 12 film excerpts were presented. Ten of the clips were selected from a validated sample of emotion-eliciting film excerpts (Schaefer et al., 2010), and the other two were added in order to obtain more specific and time-locked emotional reactions (for a complete list of selected videos, see the Supplementary Material—Section A).

The duration of each clip ranged from 1 to 4 min, and, according to Schaefer et al. (2010), each of them elicited to a greater extent the following specific emotion: disgust, happiness, anger, sadness, and fear.

Participants were instructed to watch the film excerpts and then answer 11 order-randomized questions, asking to what extent they felt happiness, anxiety, anger, calm, disgust, joy, embarrassment, fear, engagement, surprise, and sadness during the clip. Each emotion intensity was rated using an analogical visual scale ranging from 0 (=not at all) to 100 (=to a very great extent).

The movie and question presentation was controlled by the software E-Prime 2.0 (Psychology Software Tools Inc., Pittsburgh, PA, United States) with the order of video clips randomized across participants. Participants’ faces were recorded during movie observation using an HD Pro Webcam c 920 full HD 1080p (Logitech, Newark, CA, United States), which was fixed at the top of the laptop. The webcam was placed in front of the participant, so to have their face in the center of the recording area.

A white panel was placed behind the participant in order to create a uniform background. Participants were asked to pay attention to the movie, fixating the screen without covering their faces with their hands. In this way, we recorded 180 video clips of participants’ faces (12 for each one) during movie observation. From these stimuli, we then selected the videos for the main experiment belonging to the condition “other.” A manipulation check was run, confirming that each clip elicited the intended emotion to a greater extent as compared to others, thus replicating previous findings (Schaefer et al., 2010; Vergallito et al., 2018) (see Supplementary Table S1). Each “other” video was offline analyzed using FaceReader 6 software (Noldus, 2014), which automatically recognizes and codes the six basic universal facial expressions (plus a neutral state expression) with an accuracy of 89% (Lewinski et al., 2014). The aim of this procedure was to select the temporal window in which the specific emotion was maximally expressed. Indeed, the software allows videos (or pictures) to be analyzed and coded frame-by-frame, producing a summary table in which emotions are expressed on a scale from 0 to 1, where 0 corresponds to the absence of emotion indexes in the facial expression and 1 indicates maximum intensity (for a description of the algorithm used by FaceReader, see van Kuilenburg et al., 2005). This procedure allowed the exact time at which participants maximally expressed a specific emotion to be selected in order to cut 3-s time-window clips from each video (for this procedure, we used video editing software, namely, Windows Movie Maker). Then, we selected six videos for each participant, each one representing a discrete emotion (disgust, happiness, anger, sadness, fear, or a neutral condition), for a total of 90 clips.

#### Phase 2: Stimuli Validation

##### Participants and procedure

Fifteen Caucasian volunteers (five males; M age = 22.7, range 21–25), naïve to the purpose of the study, took part in the experiment in exchange for course credits.

Participants sat in an artificially illuminated room at 60 cm from a 15.7” laptop monitor, where the 90 video clips previously created were presented using the E-Prime 2 software. After each video, participants were asked: (i) to rate the valence of the actor’s facial expression on an analogical visual scale ranging from 0 (negative) to 100 (positive); (ii) to select the specific emotion conveyed by the actor’s facial expression among seven alternatives (disgust, happiness, anger, sadness, fear, neutral, none of the previous options); (iii) to rate how much they felt confident about their choice (0 = not confident at all, 100 = confident to a very great extent); (iv) how intense the emotion expressed by the facial expression was (0 = not intense at all, 100 = very intense); (v) how aroused they felt aroused during the video presentation (0 = not aroused at all, 100 = very aroused). We selected the three participants whose emotions, conveyed by facial expressions, were most accurately identified by the fifteen judges. In this way, we obtained six clips for each participant, each one representative of a specific emotion, for a total of 18 clips, which were used as videos belonging to the *other-expression condition* in the main experiment (see Supplementary Table S2 for the judges’ ratings).

### Main Experiment: EMG Recordings

#### Participants and Procedure

Eighteen healthy volunteers (15 females and three males, M age = 22.4, range = 19–26) participated in the study. Participants were Caucasian, right-handed, and had normal vision or contact lens-corrected vision; males were shaved and without mustache. All participants were naïve to the purpose of the study. Participants took part in a two-session experiment. The first session aimed at collecting video of the participants’ depicting their own facial expressions (*self-expression condition*). The second session represented the core of the present research project, consisting of facial EMG recording during the view of *self* and *other* videos.

#### First Session Procedure: Self-Stimuli Creation

The first session procedure was the same as phase one of the preliminary experiment (see section “Phase 1: Stimuli Preparation”), summarized as follows: (i) participants watched film excerpts, during which we recorded their faces; (ii) the videos were analyzed using FaceReader Software in order to select the time-window at which a given emotion was maximally expressed; (iii) for each of the six discrete emotions, a 3-s video was cut from the entire registration, thus obtaining six videos for each participant.

#### Second Session Procedure: Facial EMG Registration

**FIGURE 1:**
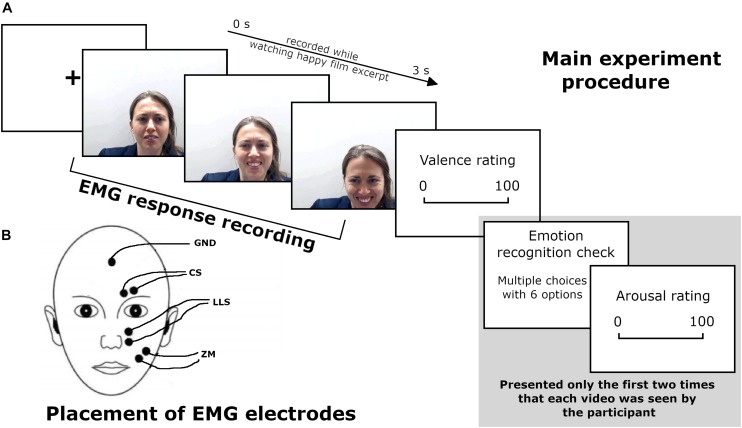
**(A)** Main experiment procedure during the EMG recording. **(B)** Schematic representation of EMG electrode placement. *Written informed consent was obtained from the participant for the publication of this image.*

The second session took place 1 week after the first one. Participants sat in a comfortable chair, in an artificially illuminated room at 60 cm from a 15.7” laptop monitor. Videos depicting their own facial expressions (*self-expression condition*) and videos of emotional expressions of three actors (*other-expression condition*) were presented during EMG recording (see Figure 1 for a scheme of the procedure). Stimuli were randomly presented using E-Prime 2 software. Each video was presented 20 times, for a total of 480 trials divided into four blocks of 120 trials each. The total number of stimuli comprised 120 trials from each of the four expressers, namely, the participant—who changed for each of the 18 subjects—and the three actors chosen in phase 1.

#### Second Session Procedure: Explicit Measure

Following each video, participants were asked to rate the valence of stimuli on an analogical visual scale from 0 (negative) to 100 (positive). Moreover, we assessed participants’ accuracy in recognizing the specific emotion and the arousal induced by each video. In particular, the first two times that a given video was presented (for a total of 48 trials), participants were asked to indicate which emotion was conveyed in a multiple-choice question with six options: (disgust, happiness, anger, sadness, fear, and neutral) and to what extent they were aroused by the video (analogical visual scale from 0—not at all to100—to a very great extent).

#### Electromyographic Recordings and Pre-processing

Facial surface EMG was recorded from three pairs of 4-mm diameter surface Ag/AgCl active electrodes corresponding to three distinct bipolar montages using a Digitimer D360 amplifier (Digitimer Ltd., Welwyn Garden City, Hertfordshire, United Kingdom). Electrodes were filled with Ten20 conductive paste (Weaver and Co., Aurora, CO, United States) and attached over the left^2^ ZM, CS, and LLS, in accordance with guidelines from Fridlund and Cacioppo (1986, see also Cattaneo and Pavesi, 2014 for an overview of facial muscle anatomy). The ground electrode was placed at the midline, at the border of the hairline (Van Boxtel, 2010). The EMG signal was recorded by a computer using SIGNAL software (Cambridge Electronic Devices, Cambridge, United Kingdom) with online filters set at 50 Hz and 2 kHz, and a sampling rate of 5 kHz. Offline, signals were then digitally band-pass filtered between 20 and 400 Hz (van Boxtel, 2001) with SIGNAL software and were segmented into 15 time-bins of 200 ms, in addition to a 500-ms pre-stimulus baseline. The magnitude of the EMG signal was computed by calculating the root-mean-square (RMS) over 200-ms time-bins after the onset of each video. Trials in which the mean RMS was greater than three standard deviations from the mean value of that specific muscle were rejected. The RMS of each trial and bin was then divided by the baseline RMS. Finally, trials were averaged for each muscle based on emotion and self/other condition.

### Statistical Analysis

Data analysis was carried out in the statistical programming environment R (R Development Core Team, 2013), using a linear mixed-effects model as the statistical procedure (Baayen et al., 2008).

For behavioral data, valence and arousal ratings were submitted to a series of linear mixed-effects regressions using the LMER procedure, whereas accuracy was submitted to a series of binomial logistic regression using the GLME procedure in the lme4 R package (version 1.1-17; Bates et al., 2015).

As fixed effects, emotion (factorial, six levels), self/other expression (factorial, two levels: self, other), bin (factorial, 15 levels^3^), and their interactions were tested with a series of likelihood ratio tests (LRT) to assess the inclusion of the effects, which significantly increased the model’s goodness of fit (Gelman and Hill, 2006). See Supplementary Tables S3–S5 for the model selection. Concerning the random effect structure, by-subject and a by-video^4^ random intercepts were included; moreover, random by-subject and by-video random slopes for emotion and self/other expression random effects were included only when the model’s goodness of fit increased.

For EMG data, pre-processed ZM, CS, and LLS activity was submitted to a series of linear mixed-effects regressions using the LMER procedure, testing in a forward stepwise LRT procedure the fixed effects of emotion (factorial, six levels), self/other expression (factorial, two levels) and bin (factorial, 15 levels), and their interactions. The random-effects structure included by-subject, by-video, and by-trial intercepts. After having fitted the full model for each muscle, influential outliers were removed via model-criticism (2.5 SD of standardized residuals).

The results of the LRT procedures for model selection and the parameters of the final best-fitting models are reported in Supplementary Tables S6–S8. *Post hoc* procedures on the final best-fitting model, applying Bonferroni correction for multiple comparisons, were carried out for direct pairwise contrasts on significant main effects and interactions using the “phia” R package (version 0.2-1, De Rosario-Martinez, 2015). See Supplementary Material—Section C for tables summarizing average and standard error mean values for explicit and implicit measures.

## Results

### Explicit Measure Results

#### Accuracy Ratings

**FIGURE 2:**
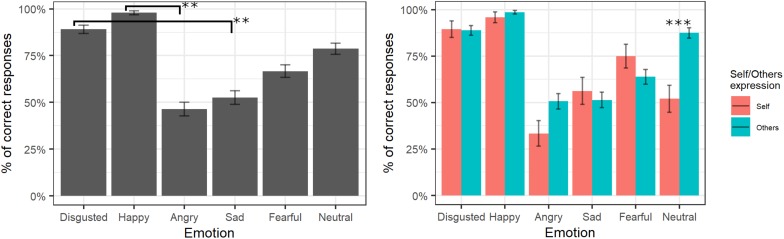
Percentage of correctly identified emotions in the main experiment. Significant differences between emotions are highlighted with asterisks (left panel, **<0.01). Percentage of correct identifications for self/other expression are depicted on the right panel, with asterisks highlighting self vs. other differences (***<0.001).

The final model of logistic regression on accuracy included the fixed effects of emotion [χ^2^(5) = 228.88, *p* < 0.001], self/other expression [χ^2^(1) = 2.38, *p* = 0.12], and their interaction [χ^2^(6) = 30.08, *p* < 0.001] (see Supplementary Table S3 for the model selection). *Post hoc* tests on the main effect of emotion showed that videos showing happy expressions were better recognized than videos showing angry expressions (*p* = 0.007), and videos representing disgust expressions were better-recognized than videos showing sad expressions (*p* = 0.006). The significant interaction showed that self/other expressions differently affected accuracy in specific emotions; in particular, *post hoc* comparisons revealed higher accuracy for other than self faces only for the neutral expressions (*p* < 0.001; see Figure 2).

#### Valence Ratings

**FIGURE 3:**
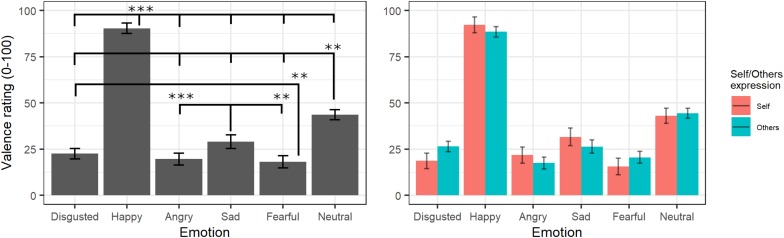
Estimated marginal means for Valence ratings of videos in the main experiment. Vertical bars represent standard error. Significant differences between emotions are highlighted with asterisks (left panel, **<0.01, ***<0.001). Valence ratings for self/other expression are depicted on the right panel.

The final model for valence ratings included the fixed effects of emotion [χ^2^(5) = 10872, *p* < 0.001] and self/other expression [χ^2^(1) = 0.001, *p* = 0.97] as well as their interaction [χ^2^(6) = 162.01, *p* < 0.001] (see Supplementary Table S4 for the model selection). *Post hoc* tests on the main effect of emotion highlighted that videos showing happy expressions were rated as more positive than videos showing all other expressions (all *p*s < 0.001) and neutral videos as more positive than those showing angry, sad, fear, and disgust facial expressions (all *p*s < 0.001). Moreover, videos showing sad expressions were rated as more positive than videos displaying anger (*p* < 0.001) and fear (*p* = 0.004), and videos showing a fear expression were rated as more negative than videos representing disgust (*p* = 0.02). The significant interaction showed that self/other expressions differently affected emotion valence ratings; however, *post hoc* tests showed no significant differences between self and other videos in any of the specific emotions (*p* > 0.05; see Figure 3).

#### Arousal Ratings

**FIGURE 4:**
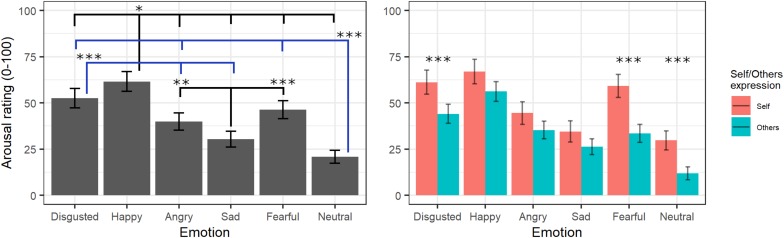
Estimated marginal means for Arousal ratings of videos in the main experiment. Vertical bars represent standard error. Significant differences between emotions are highlighted with asterisks (left panel, *<0.05, **<0.01, ***<0.001). Arousal ratings for self/other expression are depicted on the right panel, with asterisks highlighting self vs. other differences (***<0.001).

The final model on arousal ratings included the fixed effects of emotion [χ^2^(5) = 508.73, *p* < 0.001] and self/other expression [χ^2^(1) = 6.04, *p* = 0.014] as well as their interaction [χ^2^(5) = 21.27, *p* < 0.001] (see Supplementary Table S5 for the model selection). *Post hoc* tests on the main effect of emotion evidenced greater arousal scores for videos showing happy expressions compared to those showing all other emotions (*p*s < 0.02), whereas neutral videos were rated with lower arousal scores compared to those showing all other emotions (*p*s < 0.001) except for sad (*p* = 0.053). Moreover, videos showing expressions of disgust were given greater arousal scores than videos with angry and sad (*p* < 0.001) expressions, and sad videos were rated as less arousing than videos with angry (*p* = 0.001) and fearful (*p* < 0.001) expressions. Parameters for the interaction effects showed that the self/other conditions differently affected arousal scores in specific emotions. Indeed, *post hoc* tests showed significantly higher arousal scores for self compared to others’ facial expressions showing fear, disgust, and neutral emotions (*p*s < 0.001) but not with angry, happy, or sad ones (*p*s > 0.2; see Figure 4).

### EMG Results

#### Zygomatic Major Activity

**FIGURE 5:**
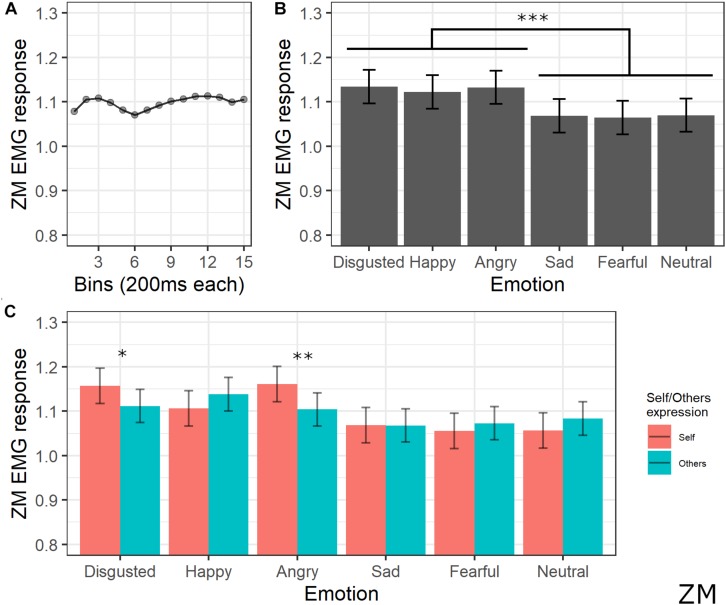
Results for ZM activity. **(A)** Main effect of bin. Each dot represents the estimated marginal mean of EMG response within the timeframe of each bin. **(B)** Main effect of emotion. Estimated marginal means of EMG response for each emotion. Vertical bars represent standard error. Significant differences between emotions are highlighted with asterisks (***<0.001). **(C)** Interaction between emotions and self/other condition. Significant differences between self vs. other are highlighted with asterisks (*<0.05, **<0.01).

The final model for ZM activity included the main effects of emotion [χ^2^(5) = 105.43, *p* < 0.001], self/other expression [χ^2^(1) = 0.52, *p* = 0.470], bin [χ^2^(14) = 28.31, *p* = 0.013], and the interaction between emotion and self/other expression [χ^2^(5) = 39.42, *p* < 0.001] (see Supplementary Table S6 for the model selection). *Post hoc* tests on the main effect of emotion revealed significant greater ZM activity for videos with happy, anger, and disgust expressions compared to videos with sad, fear, and neutral expressions (all *p*s < 0.001). *Post hoc* analyses on bin main effect showed no significant difference. Direct *post hoc* contrasts on the effect of self/other expression in each emotion showed significantly greater ZM activity for self compared to other videos when angry (*p* = 0.001) and disgust (*p* = 0.013) emotions were presented (see Figure 5).

#### Corrugator Supercilii Activity

**FIGURE 6:**
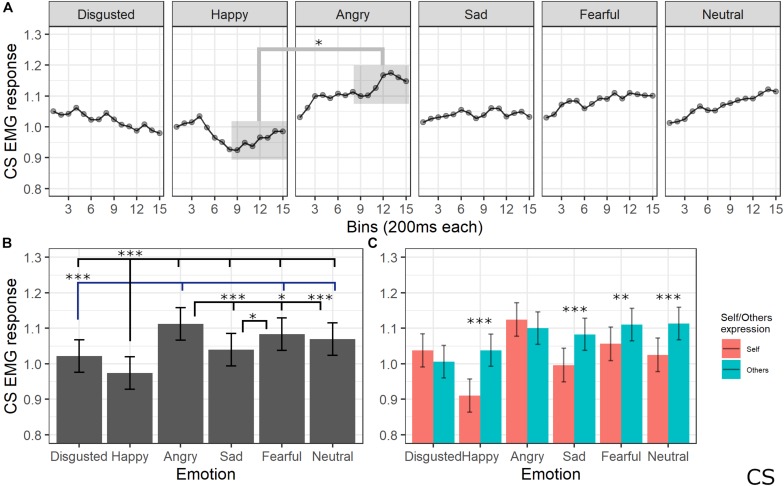
Results for CS activity. **(A)** Main effect of the self/other condition. Estimated marginal means of EMG response for video of self vs. others. Significant difference highlighted with asterisks (*<0.05). Vertical bars represent standard error. **(B)** Main effect of emotion. Estimated marginal means of EMG response for each emotion. Significant differences between emotions are highlighted with asterisks (*<0.05, ***<0.001). **(C)** Interaction between emotions and self/other condition. Significant differences between self vs. other are highlighted with asterisks (**<0.01, ***<0.001).

The final model for CS activity included the main effects of emotion [χ^2^(5) = 205.64, *p* < 0.001], self/other expression [χ^2^(1) = 63.36, *p* < 0.001], and bin [χ^2^(14) = 19.59, *p* = 0.144], as well as emotion by self/other expression [χ^2^(5) = 86.19, *p* < 0.001] and emotion by bin [χ^2^(70) = 91.83, *p* = 0.041] interactions (see Supplementary Table S7 for the model selection). *Post hoc* tests performed on the main effect of self/other expression showed that CS activity was lower for self compared to other videos (*p* < 0.001). *Post hoc* testing performed on emotion main effect showed significantly lower activity of CS for videos with a happy expression compared to all emotions (*p*s < 0.001) and for disgust compared to angry, fear, and neutral expressions (*p*s < 0.001). Moreover, CS activity was significantly higher for angry compared to sad (*p* < 0.001), neutral (*p* < 0.001), and fearful (*p* = 0.029) expressions and for fear compared to sad videos (*p* < 0.001). Finally, activity was lower for sad compared to neutral videos (*p* = 0.029). Self/other expression differently affected CS activity depending on the specific emotion: *post hoc* tests revealed significantly lower activity for self compared to others’ faces when happy (*p* < 0.001), sad (*p* < 0.001), fear (*p* = 0.001), and neutral (*p* < 0.001) expressions were presented. Finally, *post hoc* on the emotion by bin interaction revealed a difference between CS activity for happy videos compared to angry ones starting from bin 6 (*p*s < 0.05; see Figure 6).

#### Levator Labii Superioris Activity

**FIGURE 7:**
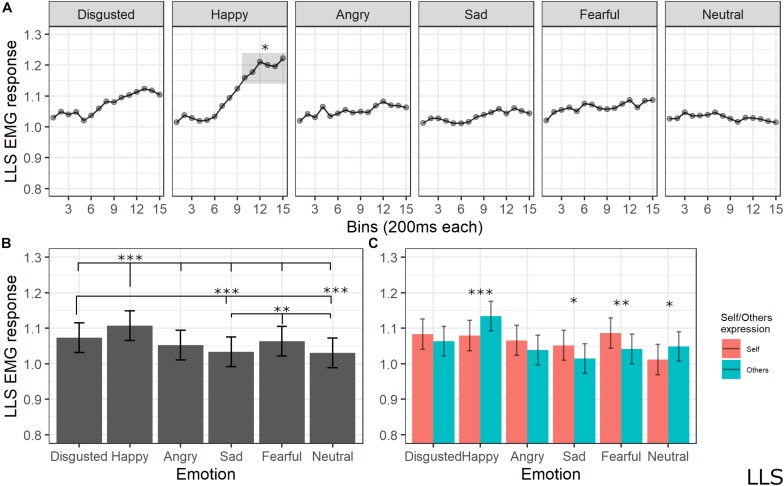
**(A)** Interaction between emotions and bin. Each dot represents the estimated marginal mean of EMG response within the timeframe of each bin. Highlighted bins are significantly higher than the same bins from other emotions (*<0.05). **(B)** Main effect of emotion. Estimated marginal means of EMG response for each emotion. Significant differences between emotions are highlighted with asterisks (***<0.001, **<0.01). **(C)** Interaction between emotions and self/other condition. Significant differences between self vs. other are highlighted with asterisks (***<0.001, **<0.01, *<0.05).

The final model for LLS activity included the main effects of emotion [χ^2^(5) = 183.1259, *p* < 0.001], self/other expression [χ^2^(1) = 1.6476, *p* = 0.19], and bin [χ^2^(14) = 117.8613, *p* < 0.001], as well as emotion by self/other expression [χ^2^(5) = 53.7085, *p* < 0.001] and emotion by bin [χ^2^(70) = 154.7846, *p* < 0.001] interactions (see Supplementary Table S8 for the model selection). *Post hoc* tests on the main effect of emotion showed significant higher LLS activity for happy expressions compared to all emotions (*p*s < 0.001, except for disgust with *p* = 0.002), for disgust compared to sad (*p* < 0.001) and neutral (*p* < 0.001) expressions, and for fear compared to sad (*p* = 0.005) and neutral (*p* = 0.001) emotions. *Post hoc* analyses of interaction between the emotion and self/other expressions showed that LLS was significantly more activated when seeing others’ compared to self faces expressing happiness (*p* < 0.001) and neutral (*p* = 0.014) emotions; higher LLS activity for self compared to others emerged for sad (*p* = 0.014) and fear (*p* < 0.01) expressions. Finally, *post hoc* testing on bin main effect revealed higher activity in later bins of the videos (from 2000 to 3000 ms) compared to the earlier bins (from 0 to 1200 ms) (*p*s < 0.05); in particular, analysis on the emotion by bin interaction revealed that LLS activity increased for happy videos compared to all presented emotions starting from bin 10 (*p*s < 0.05; see Figure 7).

## Discussion

The present study aimed at exploring, for the first time, the neurophysiological and cognitive underpinnings of SMT by assessing explicit and implicit responses to the view of own vs. others’ faces dynamically expressing discrete emotions. Ecological dynamic stimuli representing the category “other” were created in a preliminary experiment by video-recording participants while watching film excerpts eliciting sad, angry, fearful, happy, disgusted, or neutral emotions. The same procedure was used in the main experiment to create “own” stimuli. Then, in the main experiment, explicit and implicit measures were collected. Explicit measures concerned emotion identification, valence, and arousal ratings, whereas facial EMG activity (implicit measure) was recorded from the ZM, CS, and LLS as measurements of facial mimicry induced by perceiving own and others’ emotional expressions.

Results on explicit measures confirmed the validity of our stimuli, which were recognized and rated for valence and arousal according to the previous literature on emotion processing. Indeed, happiness was the emotion with the highest accuracy score, confirming a longstanding finding on the advantage of happy-face recognition over all negative facial expressions (Feyereisen et al., 1986; Kirita and Endo, 1995; Leppänen and Hietanen, 2003; Palermo and Coltheart, 2004; Juth et al., 2005; Calvo and Lundqvist, 2008; Tottenham et al., 2009; Calvo et al., 2010), likely due to its highly salient and distinctive facial features. Interestingly, an interaction between emotion and the self/other condition was found, consisting of better accuracy in recognizing other compared to own neutral emotion, which is considered the most ambiguous facial expression. Crucially, 34.7% of participants categorized their own neutral face as representing an emotion, which was negative in 88% of the incorrect categorizations (sadness in 73%, fear in 9%, and angry in 18% of trials), while it was rated as positive (happiness) in 12% of trials. Only in 15.3% of trials did participants evaluate others’ neutral faces as depicting an emotion. The tendency to attribute the status of emotion even to a minimum sign of muscular activation in own faces can be interpreted in the light of the embodied simulation theory and is a well-known and frequent effect in the clinical application of SMT. Clinical experience with SMT, indeed, suggests that the patient recognizes an emotion on his/her face even when highly expert therapists are not able to detect it (Vinai et al., 2016). When requested to pick the frame, patients are incredibly competent at indicating on the screen the exact moment in which they see the minimal movement of the lips or of the eyes indicating the emotional state.

Concerning valence ratings, as expected, happiness was the most positively rated expression, followed by neutral, sadness, disgust, anger, and fear, which received lower ratings. From a clinical perspective, it is interesting that sadness was not considered the most negative expression. This result is in line with previous studies: indeed, the expression of sadness—as happiness—signals an affiliative intention of the expresser, inducing an increase of liking, prosocial behavior, and other positive actions (Jakobs et al., 2001; Hess and Fischer, 2013, 2017).

Concerning arousal ratings, happiness in the *own condition* was rated as the most arousing condition, whereas neutral *other* was rated as the lowest. Interestingly, own expressions were rated as more arousing overall, with fear, disgust, and neutral expressions reaching a statistically significant difference as compared to the other condition. It is of note that the observation of own sadness was less arousing as compared to the other negative expressions (and less negatively rated), in line with clinical consideration. Indeed, patients’ observation of their own face while recalling sad life events typically induces a positive sense of self-compassion (Vinai et al., 2015; Petrocchi et al., 2017). In the light of the attachment theory (Ainsworth and Bowlby, 1991), we can speculate that the sight of ourselves in trouble induces a positive desire of caregiving (more than desperation), which would be useful from an evolutionary point of view.

Regarding implicit measures, the EMG pattern that emerged in our study is in line with previous research concerning the muscular-specific activation induced by emotion valence, thus confirming the validity of our experimental apparatus. Indeed, CS activity was maximally increased by angry faces and reduced during the observation of happy expressions (e.g., Dimberg and Thunberg, 1998; Dimberg and Petterson, 2000; see Hess and Fischer, 2013 for a review). This pattern is the most consistently reported when analyzing facial EMG, suggesting that the CS tenses during negative emotion processing and relaxes during processing positive emotions (Ekman, 2007). Moreover, our results on explicit measures suggested that the expression of anger (together with fear) was rated as more negative compared to the other emotions. ZM activity was higher for stimuli depicting happiness, in line with previous studies suggesting the involvement of this muscle during the processing of positive valence stimuli (e.g., Larsen et al., 2003; Tan et al., 2012; Hess and Fischer, 2013). LLS activity was maximally increased by the expression of happiness as compared to all other emotions and of disgust as compared to sadness and neutral emotions. In line with our findings, LLS activity has been reported to be specifically involved in the expression and facial mimicry of disgust (Lundqvist and Dimberg, 1995; Van Boxtel, 2010), but some previous studies also found an increased activity of this muscle during the processing of positive valence emotions (Lundqvist, 1995; Vergallito et al., 2019).

Crucially, the emotion expressed in the videos interacted with the self-other condition in influencing EMG activation. The CS was more relaxed during the observation of own expressions of happiness, sadness, fear, and the neutral condition as compared to the same emotions expressed by others. As previously mentioned, the typical pattern of CS activation consists of reducing its activity during positive emotions; indeed, it seems reasonable that processing one’s own emotion of happiness induces a stronger effect, namely, a lower activation. Less clear is which mechanisms reduce CS even for other emotions that are considered as negative. A possible speculation, based on the clinical evidence previously discussed (Vinai et al., 2015), is that the reduced activity of the CS might reflect a positive feeling of self-compassion induced by observation of one’s own emotion. For the ZM, the interaction between emotion expression and the self vs. other condition was significant during the observation of angry and disgusted faces, with higher activity for the own condition as compared to the other condition. To the best of our knowledge, no previous studies indexed the mimicry of these emotions to the ZM; thus, further studies are needed to account for this dissociation. Finally, in the LLS, we found higher activation for the other condition as compared to the self condition for happy and neutral expressions, while increased activity in the self condition emerged for sadness and fear expressions.

These results contrast with the hypothesis that facial mimicry is fully automatic, as predicted by the Motor Match Hypothesis since, if the process were simply based on a perception–behavior link, it should be independent of who is the expresser and who is the perceiver. Our results suggest instead that EMG recorded activity also reflected a post-interpretative stage, corresponding to the emotion experienced as a consequence of the one observed on the expresser’s face.

Despite the specific pattern of ratings and muscular activations found in the present work, the main result that clearly emerged is a dissociation at both explicit and implicit levels in emotional processing of self vs. other emotions. Few studies have systematically investigated this issue. What we know from past research is that self-related stimuli are more relevant to us than those related to others (e.g., Ross and Sicoly, 1979; Brédart et al., 2006) and that this sense of self is intrinsically linked to one’s own face (e.g., Porciello et al., 2014). Crucially, even though we can perceive our face only by looking at a mirror, the view of our own face is more effective in activating mirror neurons than is perceiving the face of another person (Uddin et al., 2005, 2007). Differences in processing self vs. other expressions have also been detected at the multisensory integration level: indeed, viewing their own face being touched modulates participants’ tactile experience more strongly than viewing other participants being touched (Serino et al., 2008).

To the best of our knowledge, no previous studies directly investigated facial mimicry occurring while viewing own as compared to others’ facial expressions. Considering our data and previous research, however, it is possible that at least two different mechanisms form the basis of STM efficacy in clinical settings. On one side, we have an automatic and more primitive facial mimicry process, which elicited in the observers the activation of the mirror neuron system and thus the previously described embodied simulation phenomenon, fostering own emotion recognition via this sensorimotor simulation. This process could be useful from an adaptive point of view: indeed, simulation helps one to understand what the other person is thinking and prepare an appropriate behavioral response. At the same time, we know from previous studies that facial mimicry is not only simulation (i.e., smiling when observing another person smiling) but can also be reactive (i.e., expressing fear when observing an angry face; Dimberg, 1982; McIntosh et al., 2006). In line with this evidence, viewing ourselves suffering might induce a different type of emotion, such as a feeling of self-compassion (Petrocchi et al., 2017). Indeed, the fact that observing own negative emotions causes greater arousal and modulation of electrophysiological responses might be the basis for a deeper comprehension and self-compassion, leading patients to recognize their suffering through the emotions depicted on their faces and becoming more aware of their emotional experience, thus providing hints on the neurophysiological mechanism at the root of SMT success.

It must be said that our findings are more in line with this second interpretation, but we ran this exploratory study on healthy participants, in which self-reflective and introspective abilities are expected to be less compromised as compared to patients.

Finally, it is possible that in our experimental participants considered their own videos differently from those showing others. Indeed, they had contextual information that made own expressions different from those of others; namely, they remembered how and when their expressions were produced. However, we do not think this possibility undermines our results: indeed, it would reinforce the idea that facial mimicry occurs only secondarily to an automatic-interpretative stage. Moreover, this would make our paradigm even more similar to the clinical setting: indeed, patients know how and when the recalled experience took place.

Taken together, our results open new avenues for future research on the contrast between explicit and implicit reactions to facial expressions and on the difference between processing own vs. others’ emotions.

### Study Limitations

The main limitation of the study is the unbalanced number of males and females (3 vs. 15) in our sample. Indeed, it is known that females are more facially expressive than males in emotion-evoking situations (e.g., Buck et al., 1972; Buck, 1984). This difference translates to larger facial muscular activity in females (e.g., Schwartz et al., 1980; Dimberg and Lundquist, 1990), which is present from childhood (Cattaneo et al., 2018) and according to previous researchers may be valence-dependent, with females being more reactive to positive emotional stimuli and males to negative ones (McDuff et al., 2017). Moreover, gender differences have also been suggested in explicit face processing, with females evaluating human faces as more positive and arousing as compared to men (e.g., Proverbio, 2017).

## Data Availability Statement

The dataset generated from the current study is available as Supplementary Material.

## Ethics Statement

The study was approved by the Ethical Committee of the Department of Psychology of the University of Milano-Bicocca, and participants’ ethical treatment was in accordance with the principles stated in the Declaration of Helsinki. Written informed consent was obtained from the individual(s) for the publication of any potentially identifiable images or data included in this article.

## Author Contributions

MS, PiV, LR, AV, and EG conceived and designed the study. VR, SA, and EL run the experiment. GM performed statistical analysis. GM, LR, and AV interpreted results. AV, GM, and LR drafted the paper, which was critically revised by all authors.

## Conflict of Interest

The authors declare that the research was conducted in the absence of any commercial or financial relationships that could be construed as a potential conflict of interest.

## Footnotes

1 Given the purpose of the present experiment, we will focus on EMG studies.

2 Muscular activity was recorded only from the left side of the face, which is more involved in emotional expressions (e.g., Rinn, 1984; Dimberg and Petterson, 2000).

3 Due to the ecologic and dynamic nature of our face recording, the maximal point of emotion expressions was not time locked across emotions and actors. Therefore, the notion of bin was reasoned to consider potential effects due to the different time points at which different emotion expressions might occur at the maximum intensity.

4 Here we referred to video to indicate each single stimulus presented to participants.
