# Supplementary material for: Explicit and Implicit Responses of Seeing Own vs. Others’ Emotions: An Electromyographic Study on the Neurophysiological and Cognitive Basis of the Self-Mirroring Technique
Source: Front Psychol. 2020 Mar 31;11:433. doi: 10.3389/fpsyg.2020.00433 (PMC7136519; doi:10.3389/fpsyg.2020.00433)
Supplement: Supplementary file 1 [file Table_1.docx]

**Supplementary Materials**

**Section A - Movies excerpts showed in the experiments**

**Happiness**

*A fish called Wanda* duration 2’56’’

*Benny and Joon*  duration 2’04’’

**Sadness**

*The Lion king* duration 2’03’’

*Dangerous minds*  duration 2’09’’

*Dead Poets Society (1)* duration 4’19’’

**Fear**

*The blair witch project* duration 4’04’’

*Shining* duration 4’20’’
*Clip 12* duration 2’34’’: this clip is a scene from the movie “*The legend of Bagger Vance*” to which a jump scare clip unrelated to the movie has been added to induce a sudden and intense fearful emotion.

**Anger**

*Schindler’s list (2)* duration 1’59’’

*Sleepers* duration 2’21’’

**Disgust**

*Trainspotting (2)* duration 1’33’’

*Seven (3)* duration 3’19’’

Table S1. Mean values of emotion intensity induced by movie excerpts in the preliminary experiment.

| Film excerpts |  | *Emotions* | | | | | | | |
| --- | --- | --- | --- | --- | --- | --- | --- | --- | --- |
|  |  | **Happiness** | **Anxiety** | **Anger** | **Calm** | **Disgust** | **Joy** | **Fear** | **Sadness** |
|  | **Benny & Joon** | **84.5 (±14.8)** | 6.9 (±9.3) | 10.4 (±22.8) | 63.5 (±32.8) | 4.2 (±7) | **80.3 (±18.2)** | 4  (±7.2) | 4.5 (±7.7) |
|  | **The Blair Witch Project** | 6.9  (±7.3) | 69.6 (±26.8) | 6.7 (±8.3) | 17.3 (±13.5) | 12.4 (±12.2) | 6.5 (±7.8) | **59.5 (±37.6)** | 13.9 (±12.3) |
|  | **The Lion King** | 3.7  (±6.2) | 28.7 (±21.5) | 28  (±30) | 40  (±23) | 7.5 (±9.1) | 4.2 (±6.7) | 16.5 (±20.8) | **90 (±11.6)** |
|  | **Dead Poets Society** | 4.9  (±6.8) | 57 (±34.1) | 26.4 (±27.7) | 26 (±19.2) | 21.4 (±31.3) | 9.7 (±21.4) | 26.4 (±29.1) | **70.7 (±26.2)** |
|  | **Dangerous Minds** | 6.5  (±7.7) | 20.1 (±19.1) | 20.1 (±22.3) | 40.7 (±27.6) | 13.3 (±13.6) | 4.9 (±6.3) | 10.7 (±14.4) | **66.1 (±26.1)** |
|  | **Schindler's List** | 4.4  (±7.7) | 36.3 (±25.9) | **65.7 (±28.5)** | 27.5 (±24.8) | 56.5 (±28.1) | 4.5 (±6.9) | 20.1 (±22) | 53.3 (±24.4) |
|  | **Seven** | 9.4 (±10.7) | 40.8 (±21.2) | 9.3 (±8.5) | 43.3 (±24.5) | **81.9 (±18.1)** | 8  (±8.5) | 16.4 (±13.7) | 13.8 (±19.2) |
|  | **The Shining** | 14.7 (±16.7) | 65.3 (±25.7) | 10.8 (±18.6) | 13.7 (±13.7) | 17.5 (±21.1) | 13.3 (±16.3) | **64.2 (±23.7)** | 15.6 (±18.6) |
|  | **Sleepers** | 5.5  (±7.7) | 56.6 (±24.6) | **69.3 (±29.9)** | 21 (±13.6) | **73.7 (±27.4)** | 5.5 (±7.7) | 26.6 (±26.3) | 62.9 (±26.6) |
|  | **Trainspotting** | 31.2 (±25.6) | 19.1 (±20.1) | 6.2 (±8.2) | 51.7 (±29.2) | **91.9 (±9.9)** | 23 (±19.7) | 7.3 (±8.6) | 6.3 (±9.5) |
|  | **A Fish Called Wanda** | **72.1 (±21.2)** | 12 (±14.5) | 4.6 (±7.1) | 57.9 (±33.1) | 9.8 (±17.8) | **67 (±16.2)** | 4.9 (±7.2) | 4.9 (±7.1) |
|  | **CLIP 12** | 72.1^[[1]](#footnote-1)^ (±21.2) | 12 (±14.5) | 4.6 (±7.1) | 57.9 (±33.1) | 9.8 (±17.8) | **67 (±16.2)** | 4.9 (±7.2) | 4.9 (±7.1) |

Table S1. Average and standard deviation values of emotions elicited by each movie except in the preliminary experiment (phase 1). Each video elicited the intended emotion to a greater extent as compared to others (except Sleepers, that induced both anger and disgust). Higher ratings are bold in the table.

Table S2. Preliminary experiment – stimuli validation.

|  | *Number of judges (n=15) identifying the emotion conveyed by participants’ facial expression.* | | | | | |  |
| --- | --- | --- | --- | --- | --- | --- | --- |
| **Participant** | **Disgust** | **Happiness** | **Neutral** | **Fear** | **Anger** | **Sadness** | **Total** |
| **6** | 15 | 15 | 13 | 13 | 10 | 15 | **81/90** |
| **14** | 15 | 15 | 11 | 12 | 13 | 9 | **75/90** |
| **11** | 11 | 15 | 14 | 10 | 13 | 6 | **69/90** |
| 12 | 13 | 15 | 13 | 9 | 7 | 10 | 67/90 |
| 4 | 14 | 15 | 13 | 6 | 8 | 6 | 62/90 |
| 15 | 12 | 15 | 6 | 13 | 4 | 8 | 58/90 |
| 1 | 15 | 14 | 5 | 14 | 1 | 8 | 57/90 |
| 3 | 0 | 15 | 11 | 11 | 5 | 12 | 54/90 |
| 8 | 12 | 14 | 5 | 6 | 5 | 12 | 54/90 |
| 2 | 2 | 15 | 15 | 14 | 3 | 2 | 51/90 |
| 7 | 10 | 15 | 10 | 10 | 2 | 4 | 51/90 |
| 16 | 2 | 15 | 14 | 4 | 5 | 8 | 48/90 |
| 9 | 9 | 8 | 12 | 5 | 10 | 3 | 47/90 |
| 5 | 6 | 15 | 10 | 7 | 3 | 2 | 43/90 |
| 13 | 2 | 15 | 10 | 2 | 8 | 5 | 42/90 |

Table S2. The table summarizes the number of judges (n=15) that correctly identified the emotion conveyed by the facial expression showed in each video. Judges recognized to a greater extent videos from the first three participants, which were used as experimental stimuli in the main experiment (total scores are bold in the table).

**Supplementary Section B – Main experiment model selection**

Table S3: Likelihood ratio tests procedure for model selection on accuracy

| *Fixed factors* | df | χ^2^ | p |
| --- | --- | --- | --- |
| Emotion | 5 | 228.88 | <.001 |
| Self/others expression | 1 | 2.3832 | =.12 |
| Emotion*Self/others expression | 6 | 30.081 | <.001 |
|  |  |  |  |
| *Random effect* |  |  |  |
| Emotion \| Subject | 21 | 86.149 | <.001 |
| Self/other expression \| Subject | 3 | 3.3852 | =.34 |
| Emotion \| Video | 21 | 116.22 | <.001 |
| Self/other expression \| Video | 3 | 0 | 1 |

Table S3 summarizes the model-simplification procedure, including degrees of freedom, chi square and significance. Fixed factors were not removed when they were part of higher order interactions (i.e. Self/others expression).

Table S4: Likelihood ratio tests procedure for model selection on valence ratings.

| *Fixed effect* | df | χ^2^ | p |
| --- | --- | --- | --- |
| Emotion | 5 | 10872 | <.001 |
| Self/other expression | 1 | 0.0012 | =.97 |
| Emotion * Self/other expression | 6 | 162.01 | <.001 |
|  |  |  |  |
| *Random effect* |  |  |  |
| Emotion \| Subject | 21 | 2067.8 | <.001 |
| Self/other expression \| Subject | NC |  |  |
| Emotion \| Video | NC |  |  |
| Self/other expression \| Video | NC |  |  |

NC = the model did not converge

Table S4 summarizes the model-simplification procedure, including degrees of freedom, chi square and significance. Fixed factors were not removed when they were part of higher order interactions (i.e. Self/others expression).

Table S5: Likelihood ratio tests procedure for model selection on arousal rating.

| *Fixed effect* | df | χ^2^ | p |
| --- | --- | --- | --- |
| Emotion | 5 | 508.73 | <.001 |
| Self/other expression | 1 | 6.0369 | =.014 |
| Emotion * Self/other expression | 5 | 21.267 | <.001 |
|  |  |  |  |
| *Random effect* |  |  |  |
| Emotion \| Subject | 21 | 92.368 | <.001 |
| Self/other expression \| Subject | NC |  |  |
| Emotion \| Video | NC |  |  |
| Self/other expression \| Video | NC |  |  |

NC = the model did not converge

Table S5 summarizes the model-simplification procedure, including degrees of freedom, chi square and significance. Fixed factors were not removed when they were part of higher order interactions (i.e. Self/others expression).

Table S6: Likelihood ratio tests procedure for model selection on ZM activity

| *Fixed effect* | df | χ^2^ | p |
| --- | --- | --- | --- |
| Emotion | 5 | 105.3697 | <.001 |
| Self/other expression | 1 | 0.5203 | =.47 |
| bin | 14 | 28.2996 | =.013 |
| Emotion * Self/other expression | 5 | 39.4206 | <.001 |
| Emotion * bin | 70 | 58.1156 | .84 |
| Self/other expression * bin | 14 | 4.5976 | .99 |
| Emotion * Self/other expression * bin | 70 | 31.4420 | .99 |

Table S6 summarizes the model-simplification procedure, including degrees of freedom, chi square and significance. Fixed factors were not removed when they were part of higher order interactions (i.e. Self/others expression).

Table S7: Likelihood ratio tests procedure for model selection on CS activity.

| *Fixed effect* | df | χ^2^ | p |
| --- | --- | --- | --- |
| Emotion | 5 | 205.639 | <.001 |
| Self/other expression | 1 | 37.751 | <.001 |
| bin | 14 | 19.581 | .144 |
| Emotion * Self/other expression | 5 | 86.046 | p<.001 |
| Emotion * bin | 70 | 91.508 | .043 |
| Self/other expression * bin | 14 | 16.278 | .296 |
| Emotion * Self/other expression * bin | 70 | 35.98 | .99 |

Table S7 summarizes the model-simplification procedure, including degrees of freedom, chi square and significance. Fixed factors were not removed when they were part of higher order interactions (i.e. Self/others expression).

Table S8: Likelihood ratio tests procedure for model selection on LLS activity.

| *Fixed effect* | df | χ^2^ | p |
| --- | --- | --- | --- |
| Emotion | 5 | 183.0662 | <.001 |
| Self/other expression | 1 | 1.6464 | 0.19 |
| bin | 14 | 117.8370 | <.001 |
| Emotion * Self/other expression | 5 | 53.6923 | <.001 |
| Emotion * bin | 70 | 154.7965 | <.001 |
| Self/other expression * bin | 14 | 4.8110 | 0.98 |
| Emotion * Self/other expression * bin | 70 | 56.0241 | 0.88 |

Table S8 summarizes the model-simplification procedure, including degrees of freedom, chi square and significance. Fixed factors were not removed when they were part of higher order interactions (i.e. Self/others expression).

**Section C – Summary tables**

Table S9: Mean and standard error of EMG activity in each condition for each muscle

|  | Emotion | ZM mean (SE) | CS mean (SE) | LLS mean (SE) |
| --- | --- | --- | --- | --- |
| Self expression videos | Disgusted | 1.158 (±0.04) | 1.037 (±0.05) | 1.083 (±0.04) |
|  | Happy | 1.107 (±0.04) | 0.91 (±0.05) | 1.079 (±0.04) |
|  | Angry | 1.168 (±0.04) | 1.125 (±0.05) | 1.066 (±0.04) |
|  | Sad | 1.066 (±0.04) | 0.996 (±0.05) | 1.052 (±0.04) |
|  | Fearful | 1.054 (±0.04) | 1.056 (±0.05) | 1.086 (±0.04) |
|  | Neutral | 1.058 (±0.04) | 1.025 (±0.05) | 1.012 (±0.04) |
| Others’expression videos | Disgusted | 1.105 (±0.04) | 1.005 (±0.05) | 1.063 (±0.04) |
|  | Happy | 1.133 (±0.04) | 1.038 (±0.05) | 1.135 (±0.04) |
|  | Angry | 1.103 (±0.04) | 1.1 (±0.05) | 1.039 (±0.04) |
|  | Sad | 1.071 (±0.04) | 1.083 (±0.05) | 1.015 (±0.04) |
|  | Fearful | 1.069 (±0.04) | 1.11 (±0.05) | 1.041 (±0.04) |
|  | Neutral | 1.08 (±0.04) | 1.113 (±0.05) | 1.049 (±0.04) |

Table S9: Mean and standard error of behavioural measures in each condition

|  | Emotion | Activation (0-100) | Valence (0-100) | Accuracy (%) |
| --- | --- | --- | --- | --- |
| Self expression videos | Disgusted | 61.14 (±6.48) | 18.66 (±4.17) | 89.6 (±4.46) |
|  | Happy | 66.9 (±6.6) | 92.24 (±4.22) | 95.8 (±2.91) |
|  | Angry | 44.53 (±6.11) | 21.7 (±4.45) | 33.3 (±6.88) |
|  | Sad | 34.49 (±5.72) | 31.59 (±4.78) | 56.2 (±7.24) |
|  | Fearful | 59.1 (±6.22) | 15.63 (±4.5) | 75 (±6.32) |
|  | Neutral | 29.71 (±5.19) | 42.95 (±4.13) | 52.1 (±7.29) |
| Others’expression videos | Disgusted | 44.07 (±5.23) | 26.39 (±2.78) | 88.9 (±2.63) |
|  | Happy | 56.14 (±5.37) | 88.43 (±2.86) | 98.6 (±0.98) |
|  | Angry | 35.25 (±4.76) | 17.47 (±3.19) | 50.7 (±4.18) |
|  | Sad | 26.22 (±4.25) | 26.35 (±3.63) | 51.4 (±4.18) |
|  | Fearful | 33.46 (±4.9) | 20.49 (±3.26) | 63.9 (±4.02) |
|  | Neutral | 11.9 (±3.5) | 44.3 (±2.73) | 87.5 (±2.77) |

1. The high value in happiness for this fearful inducing clip is explained by how the clip was made: the original scene depicts an adult teaching a young kid how to play golf, and at the end of it a clip depicting a loud scary face was added in order to induce sudden fear. [↑](#footnote-ref-1)
