# Supplementary material for: Explicit and Implicit Responses of Seeing Own vs. Others’ Emotions: An Electromyographic Study on the Neurophysiological and Cognitive Basis of the Self-Mirroring Technique
Source: Front Psychol. 2020 Mar 31;11:433. doi: 10.3389/fpsyg.2020.00433 (PMC7136519; doi:10.3389/fpsyg.2020.00433)
Supplement: Supplementary file 2 [file Table_2.pdf]

Datasets collected and analyzed in the main experiment (18 participants).

#Dataset: mirror_behav.csv or Data Sheet 1

The dataset includes participants' explicit ratings, namely valence and arousal, and emotion recognition accuracy.

Arousal and Accuracy were recorded only in the first two presentations of each video (values of other presentations are missing, we substituted each of them with NA).

NB: It's a comma separated table.

Subject = subjects' identifier

Gender = factor, participant's gender (F=female, M=male)

Emotion = factor, emotion expressed in each video

Video_actor = factor, person appearing in each video (Actors 6, 11, 14 = when other facial expressions are shown; Participant= when own facial expressions are depicted)

Selfother = factor, indicates whether the video shows self vs others facial expressions

Valence = numeric, valence rating for each video (0-100)

Arousal = numeric, arousal rating for the first two presentations of each video (0-100)

Accuracy = factor, 0 for incorrect answers, 1 for correctly recognized emotions.

#Dataset: mirror_emg.csv or Data Sheet 2

The dataset includes participant's facial EMG activity, recorded from the currugator supercilii (CS), the zygomaticus major (ZM) and the levator labii superioris (LLS) muscles.

It's a comma separated table, with . indicating decimal points.

Subject = subjects' identifier

Trial = trials' order (1-480)

Emotion = factor, emotion expressed in each video

Video_actor = factor, person appearing in each video (Actors 6, 11, 14 = when other facial expressions are shown; Participant= when own facial expressions are depicted)

Selfother = factor, indicates whether the video shows self vs others facial expressions

Bin = factor, indicates time frames of each video (1-15).

ZM = numeric, left ZM EMG activity

CS = numeric, left CS EMG activity

LLS = numeric, left LLS EMG activity
